# Sustained EGFR Signaling Expands Otx2^+^ and Chx10^+^ Retinal Progenitors in the Postnatal Mouse Retina

**DOI:** 10.3390/cells14231854

**Published:** 2025-11-25

**Authors:** Sanja Ivkovic, Tamara Major, Miroslav Adzic

**Affiliations:** 1 Vinca, Institute of Nuclear Sciences, University of Belgrade, Mike Petrovica Alasa 12-14, 11001 Belgrade, Serbia; miraz@vin.bg.ac.rs; 2 Independent Researcher, 11221 Beograd, Serbia; majtamara@gmail.com

**Keywords:** epidermal growth factor (EGF), EGFR, retinal progenitors, Otx2, Chx10, photoreceptors, bipolar cells, postnatal retina, retinal explants, proliferation

## Abstract

**Highlights:**

**What are the main findings?**

- Sustained EGF signaling during postnatal retinal development enhances progenitor proliferation, increases EGFR expression, and expands Otx2+ and Chx10+ progenitor populations.
- The postnatal retinal explant system provides a physiologically relevant model that retains the retinal architecture while enabling controlled manipulation of growth factor signaling.

**What are the implications of the main findings?**

- These findings indicate that prolonged EGFR activation stabilizes specific progenitor identities, supporting future studies aimed at defining their molecular trajectories and differentiation potential.
- The explant platform offers a practical tool for preclinical assessment of signaling modulators and regenerative strategies, bridging stem cell culture systems and in vivo models.

**Abstract:**

The regenerative potential of the mammalian retina is limited, yet identifying signaling pathways that influence progenitor cell behavior remains an important step toward understanding the mechanisms of retinal development and plasticity. Epidermal Growth Factor Receptor (EGFR) signaling has been implicated in regulating proliferation and differentiation in the central nervous system, but its role in the postnatal retina is less defined. In this study, we employed an ex vivo explant model of the postnatal mouse retina to investigate the effects of sustained Epidermal Growth Factor (EGF) stimulation. Our results demonstrate that EGF extends the proliferative activity of progenitors that are normally quiescent after birth. However, the sustained EGFR activation (10 ng/mL, for 7 days) in the postnatal retina not only promotes EGFR+ progenitor proliferation but also maintains co-expression of Otx2 and Chx10, revealing a distinct progenitor population, suggesting that extended EGF signaling influences lineage allocation. These findings indicate that EGFR activation can modulate both the maintenance and differentiation potential of retinal progenitors in a context-dependent manner. While additional studies are needed to determine whether these progenitors develop into mature, functional neurons, our work provides a framework for future investigations into signaling pathways that may be leveraged to influence retinal development and plasticity.

## 1. Introduction

The vertebrate retina develops from a pool of multipotent progenitor cells that give rise to all major neuronal and glial lineages in a temporally regulated manner. During embryonic development, these progenitors proliferate actively to establish the retinal architecture. After birth, however, the proliferative capacity of retinal progenitors rapidly declines, resulting in a largely quiescent postnatal retina [1,2,3]. Understanding the mechanisms that regulate this transition from proliferation to differentiation remains critical for elucidating how retinal growth and regenerative potential are controlled.

Epidermal Growth Factor (EGF) and its receptor (EGFR) are well-known mediators of neural progenitor proliferation and survival across multiple regions of the central nervous system, including the retina [4,5,6,7]. However, EGF’s influence on progenitor behavior in the postnatal retina is less clearly defined. This gap in knowledge limits our understanding of the molecular cues that may modulate residual proliferative competence in the maturing retina.

Recent findings indicate that subsets of postnatal retinal cells, including Müller glia and progenitor-like populations, can re-enter the cell cycle under specific stimuli or injury conditions [6,8,9]. These observations suggest that growth factor signaling may be harnessed to enhance endogenous regenerative responses. Nevertheless, the cellular outcomes and differentiation potential of progenitors responding to EGF in the postnatal retina remain incompletely understood. EGF signaling is, in general, considered to induce proliferation and antagonize differentiation in retinal precursors in vitro and in vivo [4,5,6,10]. However, EGF signaling has been shown to be involved in retinal progenitors’ cell fate decisions [4,7,11] and in the coordination of photoreceptor recruitment and specification in Drosophila [11,12,13,14]. Heparin-bound EGF (HB-EGF) is both necessary and sufficient for retinal regeneration in zebrafish [15]. In mammals, EGF’s effects have been extensively examined in Müller glia proliferation and dedifferentiation [16,17]. However, its influence on postnatal progenitors giving rise to photoreceptors and bipolar cells remains poorly characterized.

Rod photoreceptors and bipolar cells are among the last retinal cell types to complete their differentiation postnatally [1,2,18]. Otx2 is a key regulator of the photoreceptor lineage [19,20,21], and the conditional knockout of Otx2 in immature retinal progenitors causes a near-total loss of rods, cones, and bipolar and horizontal cells [21], indicating that Otx2^+^ progenitors give rise to both photoreceptors and bipolar cells. In addition, Chx10 (Vsx2) is a transcription factor essential for bipolar cell development, and its loss results in a near-complete absence of bipolar cells [22,23].

In the present study, we examine the effects of EGF on progenitor proliferation and cellular identity in the postnatal retina, employing an ex vivo retinal explant (RE) model [24]. Retinas were cultured for seven days in vitro (7 DIVs), mirroring the first postnatal week of development. We focused on the proliferative potential of EGFR^+^ cells and the expression patterns of Otx2 and Chx10 as progenitor markers. This work establishes a foundation for future studies aimed at modulating EGFR pathways to stimulate retinal repair and regeneration.

## 2. Materials and Methods

### 2.1. Animals

All animal experiments were approved by the Animal Ethics Committee of Instituto de Medicina Molecular (AEC_027_2010_DH_Rdt_general_IMM) and conducted in accordance with national regulations. C57BL/6 mice (3–4 per cage) were housed under standard conditions (23 ± 2 °C, 60–70% humidity, 12 h light/dark cycles) with free access to food and water. Newborn pups were used for P0 retinal explant preparation. Animals were cryo-anesthetized and decapitated, and their eyes were enucleated using curved forceps for processing. Experimental animals were divided into two groups: (1) control group (n = 4) and (2) EGF-treated group (n = 4). P7 animals were euthanized by CO_2_ asphyxiation for immunofluorescence analysis (n = 3). The eyes were enucleated and cryopreserved. No a priori power calculation was performed; the sample size was consistent with prior retinal explant studies and determined empirically to ensure reproducibility.

### 2.2. Retinal Explant Culture

Retinal explants were prepared as described by Hatakeyama and Kageyama [25]. Enucleated eyes were transferred to DMEM containing 50 IU–μg/mL penicillin–streptomycin. Sclera and cornea were peeled away, and the retina was isolated from the lens and vitreous. The retinal pigment epithelium (RPE) sheets were gently removed. The retinas were placed on semi-porous Millicell-CM membranes (0.4 μm pore size, Millipore, Burlington, MA, USA) with the photoreceptor layer facing the membrane surface. Explants (2–3 per filter) were transferred to six-well plates containing 1 mL growth medium per well. The control medium contained 50% MEM–Hepes, 25% Hanks’ balanced salt solution, 5.75 mg/mL glucose, 25 U/mL penicillin, 25 mg/mL streptomycin, 200 mM L-glutamine, 1× B27, and 1× N2 supplements. The medium was changed every other day. For EGF-treated cultures, 10 ng/mL EGF (Sigma, St. Louis, MO, USA) [26] was added throughout the 7 DIVs period.

### 2.3. BrdU Labeling and Immunostaining

To assess proliferation, BrdU (10 μg/mL) was added to cultures either on day 0 (first 24 h) or day 6 (last 24 h). At 7 DIVs, explants were fixed in 4% paraformaldehyde (PFA), cryoprotected, sectioned (12 μm), and processed for immunofluorescence using rat anti-BrdU antibody (1:1000, Oxford Biotechnology, Oxford, UK).

### 2.4. Immunofluorescence

Samples were fixed in 4% PFA overnight at 4 °C, cryoprotected in 30% sucrose, and embedded in 7.5% gelatin–15% sucrose. Sections (12 μm) were degelatinized at 37 °C for 15 min. For Chx10, sections were pretreated with 3% H_2_O_2_ in methanol for 30 min. Permeabilization was performed with 0.5% Triton X-100 for 15 min, followed by blocking (10% normal goat serum, 0.1% Triton X-100, Sigma, St. Louis, MO, USA) for 1 h at room temperature. Primary antibodies were incubated overnight at 4 °C. Antibodies: rabbit anti-EGFR (1:100, Abcam, Cambridge, UK), sheep anti-Chx10 (1:100, Exalpha, Vector Laboratories, Inc., Newark, CA, USA), rat anti-BrdU (1:1000, Oxford Biotechnology, Oxford, UK), goat anti-Otx2 (1:500, R&D, Minneapolis, MN, USA), rabbit anti-Otx2 (1:1000, Abcam, Waltham, MA, USA), rabbit anti-Recoverin (1:1000, Chemicon, Rolling Meadows, IL, USA), and mouse anti-Rhodopsin (1:500, DSHB, Iowa City, IA, USA). Secondary antibodies: Alexa Fluor 488/594 (1:400, Molecular Probes, Eugene, OR, USA). Nuclei were counterstained with DAPI.

### 2.5. Imaging and Quantification

Images were captured using a Leica DM5000B microscope (Wetzlar, Germany). The INL was identified by nuclear morphology (light, round nuclei). A sample size of n = 3–4 per condition was chosen based on prior retinal explant studies and our preliminary experiments, which indicated that this number was sufficient to detect robust and reproducible differences in proliferation and progenitor marker expression. Each experiment was repeated across multiple independent explant cultures derived from different litters, and consistent outcomes were observed in all replicates. Quantitative data represent the mean ± SEM across these independent cultures, ensuring that the results reflect reproducible biological effects rather than isolated observations. EGFR^+^, Otx2^+^, and Chx10^+^ cells were quantified per region of interest (ROI; 3000 μm^2^) across 4–6 fields per explant. DAPI^+^ nuclei were counted in the same ROIs. Data represent the mean ± SEM from multiple explants.

### 2.6. Statistical Analysis

Data were analyzed using Prism 7 (GraphPad Prism, Software, v.6, La Jolla, CA, USA). No exclusion criteria were specified; all explants prepared under standard conditions were included. No animals or explants were excluded from the analysis. Exact n values per group are reported in the figure legends (e.g., n = 3 or n = 4 for each condition). Mann–Whitney nonparametric tests were chosen to account for the small sample size and non-Gaussian data distribution. *p* < 0.05 was considered significant.

## 3. Results

### 3.1. Characterization of Control and EGF-Treated Retinal Explants

**Figure 1:**
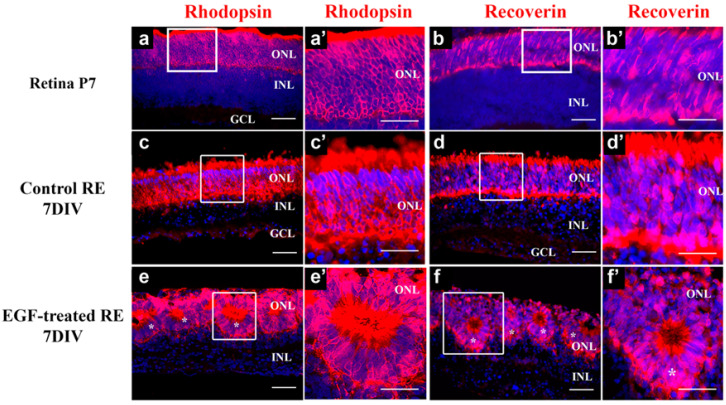
Control and EGF-treated REs at 7 DIVs recapitulate P7 in vivo retinal photoreceptor development. The expression pattern of Rhodopsin ((**a**,**a’**,**c**,**c’**,**e**,**e’**), red) and Recoverin ((**b**,**b’**,**d**,**d’**,**f**,**f’**), red) in P7 retina (**a**,**a’**,**b**,**b’**), control 7 DIVs REs (**c**,**c’**), and EGF-treated 7 DIVs REs (**e**,**e’**,**f**,**f’**). Photoreceptors in EGF-treated REs are predominantly organized as rosettes, labeled with asterisks (**e**,**e’**,**f**,**f’**). (**a’**–**f’**)—magnified boxed areas from (**a**–**f**), respectively. Dapi—blue. n = 4. Scale bars: 50 μm.

Retinal explants can be grown in culture conditions for a couple of weeks, successfully recapitulating the in vivo differentiation pattern [25]. We established an in vitro explant culture of P0 retinas as previously described (see Methods) and grew them for 7 days (7 DIVs) either in the control medium or in the presence of exogenous EGF (10 ng/mL). We selected a 10 ng/mL concentration of EGF because this dose has been shown to activate EGFR signaling in the mammalian retina and alter the progenitor content [26,27]. At this level, EGF produces robust EGFR phosphorylation and ERK/AKT activation, and these responses are fully blocked by AG1478, confirming pathway specificity [26]. Accordingly, 10 ng/mL represents a physiologically relevant and well-supported dose that avoids the potential off-target effects of higher EGF concentrations. At the end of day 7, the retinal explants (REs) had already developed the outer nuclear layer (ONL) and the inner nuclear layer (INL) (Figure 1c), similar to the developmental patterning taking place in vivo in retinas obtained from postnatal-day-7 mouse pups (Figure 1a). In the presence of the exogenous EGF, retinal lamination was also preserved, and the ONL and INL were established. However, the organization of the ONL in EGF-treated REs was altered (Figure 1e,f, Supplemental Figure S1), and the photoreceptors in the ONL were mainly organized as rosettes. In order to establish how this irregular organization of the ONL affected the development of photoreceptors in EGF-treated REs, we stained the explants at 7 DIVs with rod-specific (Rhodopsin) and general (Recoverin) photoreceptor markers. The staining revealed clearly defined ONL layers in both control (Figure 1c,c’,d,d’) and EGF-treated REs (Figure 1e,e’,f,f’) and showed that the photoreceptors in general, and the rods among them, were differentiated, recapitulating the patterning observed in P7 retinas (Figure 1a,a’,b,b’). Thus, even with the rosette-like organization of the ONL in EGF-treated REs, the general pattern of the specific photoreceptor marker expression was maintained (Figure 1).

### 3.2. Sustained EGF Signaling Extends Progenitor Proliferation

**Figure 2:**
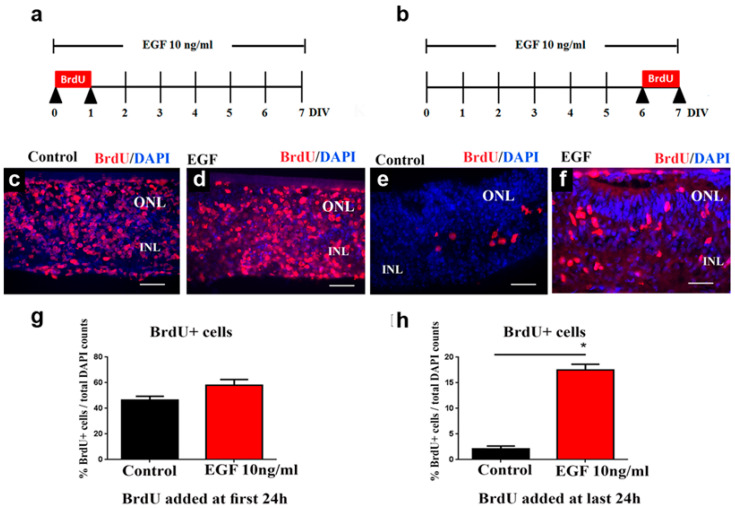
Proliferative potential of retinal progenitors is extended during sustained EGF signaling. (**a**,**b**) Schematic representation of BrdU treatment strategies. (**c**–**f**) Staining of REs at 7 DIVs with BrdU (red) and DAPI (blue). The BrdU expression pattern was similar in the control (**c**) and EGF-treated (**d**) REs at 7 DIVs when BrdU (10 μM) was added to the explant media during the first 24 h of explant culture. The number of BrdU+ cells was increased in the EGF-treated (**f**) REs when compared to the controls (**e**) when BrdU (10 μM) was added during the last 24 h of the explant culture. (**g**) Quantification (mean ± SEM) of the percentage of BrdU+ cells in the explants treated with BrdU during the first 24 h. (**h**) Quantification (mean ± SEM) of the percentage of BrdU+ cells in the explants treated with BrdU during the last 24 h. The number of BrdU+ cells is represented as the percentage of the total DAPI count. n = 3 for each condition examined. * *p* < 0.05, by Mann–Whitney test. Scale bars: 50 μm.

In order to establish if the proliferation index was changed under sustained EGF signaling, we added BrdU (10 μg/mL) to the culture medium and analyzed BrdU+ cells at 7 DIVs using anti-BrdU antibody for immunostaining. BrdU was added to the medium either during the first 24 h (Figure 2a,c,d) or during the last 24 h (Figure 2b,e,f) of the 7 DIVs treatment, to both control (Figure 2c,e) and EGF-treated (Figure 2d,f) REs. The quantification of BrdU+ cells showed that the proliferation index was not altered in EGF-treated REs if BrdU was added during the first 24 h of the 7-day treatment (46.5% vs. 57.9%, respectively, Figure 2g). However, if BrdU was added during the last 24 h of the 7-day EGF treatment, the number of BrdU+ cells was significantly increased (8-fold) in the EGF-treated REs when compared to the control REs (from 2.075% in control REs to 17.46% in EGF-treated REs, Figure 2h). These results indicate that in the presence of continuous EGF signaling, the late retinal progenitors, at the end of the first postnatal week, which are normally quiescent at that time, are still able to activate their proliferative potential.

### 3.3. EGF Treatment Increases EGFR^+^ Cell Populations

**Figure 3:**
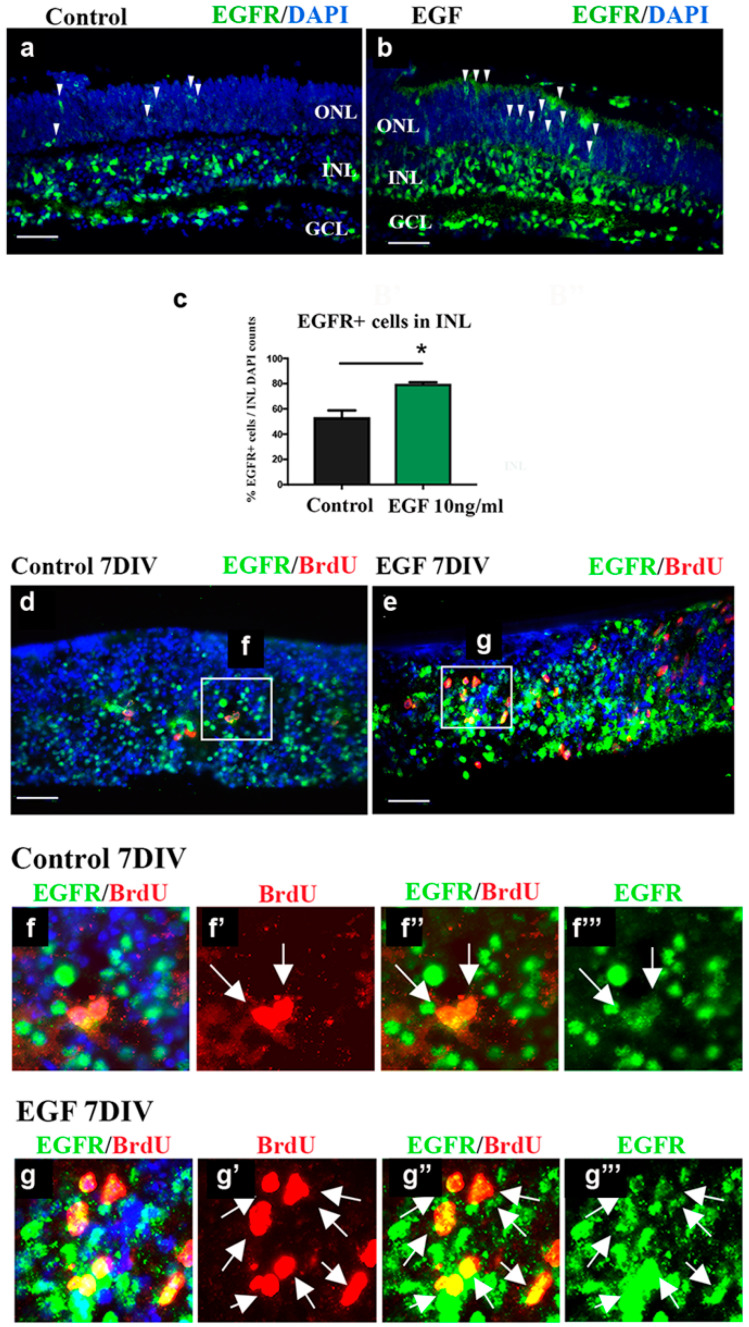
EGFR+ cells in postnatal retina continue to proliferate in the presence of exogenous EGF at 7 DIVs. (**a**,**b**) Control (**a**) and EGF-treated (**b**) REs stained with EGFR (green) and DAPI (blue). Arrowheads—EGFR+ cells (**c**) Quantification (mean ± SEM) of the EGFR+ cells per ROI in the INL showed an increase in the EGF-treated REs when compared to the controls (12.1 vs. 7.7, respectively) at 7 DIVs. (**d**) The number of DAPI+ cells counted in the same ROIs did not show any statistically significant differences between the control and EGF-treated REs. (**e**–**g**) Staining of the EGF-treated REs with BrdU (red), EGFR (green), and DAPI (blue) revealed that EGFR+ cells were BrdU+ (arrows). n = 4 for each condition analyzed. * *p* < 0.05, by Mann–Whitney *t*-test. Scale bars: 50 μm.

The increase in the progenitor proliferation rate, observed in EGF-treated REs, prompted us to analyze the changes in the expression pattern of EGFR+ cells. In the control REs, EGFR+ cells were mainly present throughout the INL and in the ganglion cell layer (GCL) (Figure 3a).

The distribution of EGFR+ cells in the EGF-treated REs shows a similar pattern to that in the control REs (Figure 3b). However, the quantification of EGFR+ cells in the INL showed a significant increase (57%) compared to control REs (*p* < 0.05) (Figure 3c). At the same time, the number of DAPI+ cells in the same ROIs used for the counting of EGFR+ cells was the same in the control and EGF-treated REs (Figure 3d). The few randomly scattered EGFR+ cells were localized in the ONL of the control REs (Figure 3a, arrowheads). In the EGF-treated REs, an apparent increase in the number of EGFR+ cells was detected in the ONL (Figure 3b, arrowheads). However, the EGFR+ cells in the ONL mostly exhibited a membranous EGFR expression, in contrast to the EGFR expression observed in the INL which was also nuclear, as is characteristic of the highly proliferating cells [28].

We, thus, focused our analyses on the INL and progenitors therein. In order to analyze the proliferative potential of EGFR+ cells during EGF supplementation, we added BrdU (10 μg/mL) during the last 24 h of the EGF treatment. Double labeling with anti-EGFR and anti-BrdU antibodies (Figure 3d–f) confirmed that EGFR+ cells in the INL were BrdU+ and proliferating under sustained EGF signaling. The results showed that all BrdU+ cells were EGFR+ in the INL of EGF-treated REs (Figure 3d–f, arrows).

### 3.4. Sustained EGF Signaling Expands Otx2^+^ and Chx10^+^ Populations

**Figure 4:**
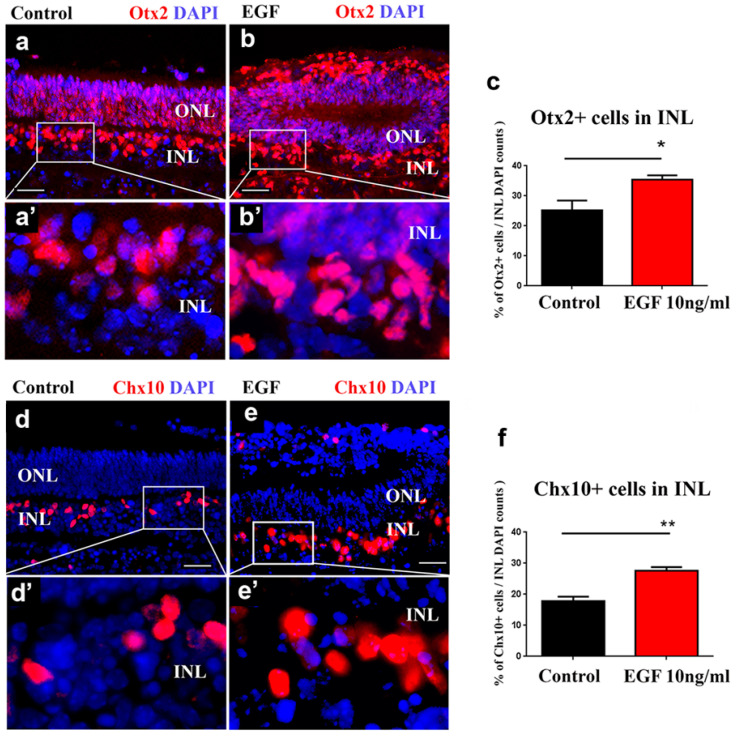
The number of Otx2+ and Chx10+ cells is increased in the INL of the EGF-treated REs at 7 DIVs**.** Staining of control (**a**,**a’**) and EGF-treated REs (**b**,**b’**) with Otx2 (red) and DAPI (blue) showed Otx2 expression in the INL and ONL in the REs at 7 DIVs. (**c**) Quantification (mean ± SEM) of the number of Otx2+ cells per ROI in the INL showed a significant (* *p* < 0.05) increase in the Otx2+ cells in the EGF-treated REs when compared to the controls (9.2 vs. 5.3, respectively). Staining of control (**d**,**d’**) and EGF–treated REs (**e**,**e’**) with Chx10 (red) and DAPI (blue) showed Chx10 expression in the INL in the REs at 7 DIVs. (**f**) Quantification (mean ± SEM) of the number of Chx10+ cells per ROI in the INL showed a significant (* *p* < 0.05, by Mann–Whitney test) increase in the EGF-treated REs when compared to the controls (8.6 vs. 6.1, respectively). The quantification of the DAPI+ cells in the same ROIs did not show any difference between the control and EGF-treated REs (**c**,**f**). n = 4 for both the control and EGF-treated REs. * *p* < 0.05, ** *p* < 0.01 by Mann–Whitney *t*-test. The scale bar is 30 μm.

As the sustained EGF signaling increased the proliferation of retinal progenitors, we wanted to establish the molecular signature of these progenitors. We analyzed the expression of Otx2+ and Chx10+, as they are markers of progenitor populations whose proliferation peaks postnatally. We first examined the expression pattern of Otx2+ cells in control REs at 7 DIVs. The Otx2+ cells were distributed in the INL but also in the ONL (Figure 4a,a’), as was reported previously [29]. A similar pattern of Otx2+ was observed in the EGF-treated REs at 7 DIVs (Figure 4b,b’). However, the number of Otx2+ cells in the EGF-treated REs in the INL was significantly increased (73%) when compared to the controls (Figure 4c). We next sought to examine the changes in the number of Chx10+ progenitors as well. The number of Chx10+ cells, localized in the INL in both the control (Figure 4d,d’) and EGF-treated REs (Figure 4e,e’), was significantly increased in the EGF-treated REs (40%) in comparison to the control REs (Figure 4f).

### 3.5. The Subpopulation of EGFR+ Cells Comprises Otx2+ and Chx10+ Retinal Progenitors

**Figure 5:**
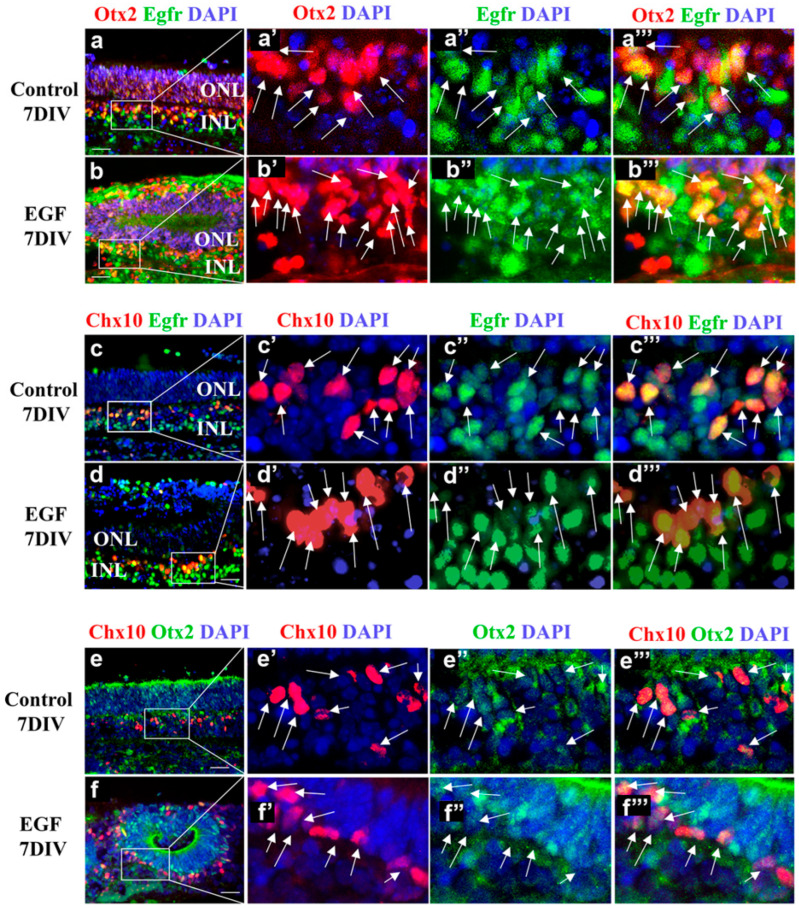
The subpopulation of EGFR+ cells is Otx2+ and Chx10+ at 7 DIVs. (**a**,**b**) Staining of the control (**a**) and EGF-treated REs (**b**) with Otx2 (red), EGFR (green), and DAPI (blue) revealed co-localization of Otx2 and EGFR staining at 7 DIVs (arrows). (**c**,**d**) Staining of the control (**c**) and EGF-treated REs (**d**) with Chx10 (red), EGFR (green), and DAPI (blue) revealed co-localization of Chx10 and EGFR staining at 7 DIVs (arrows). (**e**,**f**) Staining with Chx10 (red), Otx2 (green), and DAPI (blue) revealed co-localization of Otx2 and Chx10 at 7 DIVs (arrows). Scale bars: 50 μm. (**a’**–**f’**)—magnified boxed areas from (**a**–**f**), respectively. (**a”**–**f”**)—magnified boxed areas from (**a**–**f**), respectively. (**a’’’**–**f’’’**)—magnified boxed areas from (**a**–**f**), respectively.

The simultaneous increase in EGFR+, Otx2+, and Chx10+ progenitors under sustained EGF signaling prompted us to investigate if Otx2+ and Chx10+ cells represent subpopulations of the expended EGFR+ progenitors. Double-labeling immunohistochemistry with anti-EGFR and anti-OTX2 antibodies showed that Otx2+ cells were EGFR+ in both the control (Figure 5a) and EGF-treated REs (Figure 5b). A strong overlap in expression was observed in the INL in both control (Figure 5a’,a”,a’’’, arrows) and EGF-treated REs (Figure 5b’,b”,b’’’, arrows). Similarly, we observed a strong co-localization of EGFR and Chx10 staining in the INL of both control (Figure 5c,c’,c”,c’’’) and EGF-treated REs (Figure 5d,d’,d”,d’’’). The co-expression of Otx2+ and Chx10+ was previously reported [30], and double staining with Otx2 and Chx10 antibodies showed a clear overlap in the expression of these two progenitor markers in both control (Figure 5e,e’,e”,e’’’) and EGF-treated REs (Figure 5f,f’,f”,f’’’). Therefore, Chx10+ and Otx2+ progenitors in the INL were EGFR+ (EGF-responsive), in both control (Figure 5c’,c”,c’’’, arrows) and EGF-treated REs (Figure 5d’,d”,d’’’, arrows), suggesting that the sustained EGF signaling led to an increase in Otx2+ and Chx10+ progenitors, via the EGFR+ progenitor pool. Thus, the sustained EGF treatment not only increased progenitor proliferation but also maintained the co-expression of OTX2 and CHX10, indicating the presence of a distinct postnatal progenitor population rather than a simple extension of cell cycle activity. Figure 5 illustrates the consistent spatial co-localization of EGFR with OTX2^+^ and CHX10^+^ progenitor populations across replicates, serving as qualitative validation of marker overlap rather than a quantitative comparison among conditions.

## 4. Discussion

Different levels of EGF signaling were shown to elicit different outcomes during cell differentiation [4,13,27,31]. The EGFR expression in the postnatal retina reaches its peak at P4, then declines [6], but persists throughout adulthood [32,33]. We thus hypothesized that the amount of ligand could be the limiting factor and that the addition of exogenous EGF can alter progenitor proliferation and differentiation during postnatal retinal development. Here, we report that sustained EGF signaling during the first week of postnatal retinal development induced the proliferation of EGFR+ progenitors, enabling the expansion of specific, Otx2+ and Chx10+, neuronal progenitor populations.

Retinal explants, as an ex vivo model system, successfully recapitulate in vivo retinal maturation, and the expression of photoreceptor-specific markers (Rhodopsin and Recoverin) confirmed the formation of the ONL in the control REs. Although our study primarily focused on the proliferative response to EGF, qualitative observations indicated that EGF-induced proliferation did not overtly disrupt retinal organization or marker expression patterns, suggesting that the differentiation potential is largely preserved. Nevertheless, the addition of EGF altered the organization of the ONL, with photoreceptors predominantly organized into rosettes. The rosette-like organization reflects localized disorganization of photoreceptor nuclei, which has been reported previously under conditions altering postnatal retinal development [34]. This morphological pattern was consistently observed across all EGF-treated explants, indicating it is a reproducible effect of sustained EGF exposure. Future studies incorporating the quantitative assessment of photoreceptor and bipolar cell markers will be important to further define the impact of EGF signaling on lineage commitment.

The sustained EGF signaling increased postnatal progenitor proliferation. While early postnatal progenitors divided at a similar rate in the control and EGF-treated REs (46.5% vs. 57.9%, respectively, Figure 2), the proliferative potential of EGF-responsive progenitors declined over time. At the end of the culturing period, at 7 DIVs, only 2% of the total number of cells was BrdU+ in the control REs. However, the sustained EGF signaling increased the proliferation of late progenitors to 17.5% (8-fold increase when compared to the control REs). With nearly all BrdU+ cells being EGFR+ at 7 DIVs, in both control and EGF-treated explants, we concluded that extended exposure to EGF leads to increased EGFR+ cell proliferation in the postnatal retina. Multiple studies have demonstrated that the effects of EGF in the retina are entirely dependent on EGFR kinase activity, providing strong support that the proliferative responses we observed arise from canonical EGFR signaling. For instance, AG1478 completely abolishes the effects of 10 ng/mL EGF on rod photoreceptor responses in retinal explants, confirming full pathway dependence at this dose [26]. Additionally, EGFR inhibition using PD153035 or PD158780 has been shown to suppress Müller glia cell cycle re-entry by up to 98% and prevent ERK1/2 activation following EGF stimulation [35] Consistent with these findings, EGFR inhibition also blocks EGF-induced proliferation and EGFR/AKT phosphorylation in human ocular cells [36]. Thus, extensive inhibitor-based evidence confirms that EGF is sufficient and necessary to activate EGFR signaling and that EGF-driven effects are abolished when EGFR activity is blocked. Although we did not directly assay EGFR phosphorylation here, these well-documented inhibitor studies provide strong mechanistic validation for interpreting our results as EGFR-dependent.

In accordance with the tissue’s high proliferative activity, EGFR expression was often localized in the nucleus (Figure 3), as was previously reported [28], indicating that EGFR might serve as a transcription factor. Nuclear EGFR has also been reported in tumors, where it can act as a transcriptional regulator to promote proliferation and survival [37]. Thus, its nuclear presence suggests a potential mechanistic contribution to progenitor proliferation. Future studies examining nuclear EGFR’s function and its interaction with cell cycle regulators will be important to clarify this role. Furthermore, EGFR has been shown to bind to the cyclin D1 promoter in vivo, and this might explain why the nuclear localization of EGFR is strongly correlated with high proliferation activity [28].

We further analyzed what the molecular signature of these proliferating progenitors is. As they were mainly localized in the INL, we focused our quantitative analyses on this retinal layer. The addition of exogenous EGF induced an increase in the number of cells that belong to the two progenitor groups that reach their peak during the first postnatal week—Otx2+ and Chx10+ cells [21,29]. The increase in the number of Otx2+ progenitors in the INL under sustained EGF signaling was 40%, when compared to the control REs. A similar increase was observed in the Chx10+ population (47%). We observed the co-localization of Otx2 and Chx10 expression, as was previously reported [29], in a subgroup of progenitors in both control and EGF-treated REs. As double staining confirmed that both Otx2+ and Chx10+ progenitors were also EGFR+, we concluded that the increased pool of EGFR+ cells in the postnatal retina has the ability to produce both Otx2+ and Chx10+ progenitors. These results are in accordance with findings that animals deficient in EGFR show a decline in the number of Chx10-expressing progenitor cells in the first postnatal week [6] and imply that sustained EGF signaling is able to increase the number of Chx10+ cells either directly or through an increase in Otx2+ progenitors. Considering that ectopic Chx10 expression was shown to be essential in driving bipolar cell genesis by inhibiting rod differentiation [23], these results indicate that EGF signaling has a role in regulating their ratio during development.

Although EGF signaling has been implicated in the induction of proliferation, recent findings in the field of stem cell differentiation showed that EGF, in addition to taurine, is able to induce the formation of Rhodopsin+ cells in mesenchymal stem cell (MSC) cultures [38]. Similarly, EGF treatment of late (postnatal) retinal stem cells (RSCs) revealed that these cells are responsive to EGF stimulation and that they have conserved their multipotency, generating both early (RGC) and late (photoreceptor, Recoverin+) retinal phenotypes [39]. Furthermore, the addition of EGF, together with FGF, can increase the plastic potential of proliferating MGs, increasing their conversion into neurons in the culture conditions [40]. While Müller glia in the mammalian retina are known to re-enter the cell cycle and acquire progenitor-like characteristics in the presence of EGF, this phenomenon was mainly studied in response to injury [6,16]. However, evidence that EGF alone in the uninjured retina can induce Müller glia to become progenitor-like cells in mice is sparce. It is possible that the OTX2^+^/CHX10^+^ cells we observed may represent either a distinct progenitor population or an as-yet-uncharacterized MG-derived pathway. Further studies, including lineage-tracing experiments, will be required to determine whether these populations are entirely independent or whether there is partial overlap with the Müller glia lineage. It was suggested that EGF can affect the chromatin architecture at the regulatory element of cyclin D1 through a process involving Cre-binding protein (CBP), histone deacetylase 1 (HDAC1), and the Suv39h1 histone/chromatin remodeling complex [41]. It is possible that such mechanisms can facilitate the expression of neuronal genes in proliferating progenitors.

Our study builds on prior work demonstrating that EGF stimulates retinal progenitor proliferation [4,6,42], but it extends these findings by identifying a distinct postnatal progenitor population characterized by OTX2^+^/CHX10^+^ co-expression. Unlike previous studies, which primarily documented proliferation or Müller glia activation, we show that sustained EGF signaling in the postnatal retina not only promotes cell cycle re-entry but also maintains the expression of key progenitor identity markers. This suggests that EGF can induce or preserve a unique progenitor subtype rather than merely extend proliferation. These findings provide new insight into postnatal progenitors’ dynamics and establish a foundation for future studies aimed at exploring their lineage potential and regenerative capabilities.

While our ex vivo explant model effectively mimics early postnatal development, it lacks systemic influences such as vascular signaling and circulating growth factors present in vivo. Although controlled EGF signaling can expand retinal progenitors while preserving differentiation, it disrupted ONL organization. Optimizing EGF delivery—using transient or pulsatile treatment, potentially combined with other growth factors—could promote progenitor expansion and retinal regeneration without compromising lamination. Time-resolved studies following EGF withdrawal will be important to define conditions that support controlled proliferation and re-differentiation for functional repair. Further studies using conditional EGFR activation or inhibition in specific progenitor populations will help clarify lineage-specific effects. Time-resolved transcriptomic analyses of Otx2^+^ and Chx10^+^ cells under sustained EGF exposure would also elucidate transcriptional programs that underlie progenitor maintenance versus differentiation. Finally, exploring how transient versus continuous EGF exposure affects the timing of differentiation may provide insights into optimizing conditions for retinal repair or stem-cell-derived retinal organoid differentiation.

## 5. Conclusions

In summary, sustained EGF signaling during postnatal retinal development enhances progenitor proliferation, increases EGFR expression, and expands Otx2^+^ and Chx10^+^ progenitor populations without preventing photoreceptor differentiation. Our results underscore the role of EGF as a critical modulator of retinal progenitor plasticity, suggesting that fine control of this pathway could be harnessed to drive targeted neurogenesis and regeneration.

Moreover, the postnatal retinal explant model described here provides a physiologically relevant platform for pharmacological and regenerative studies. By preserving the retinal architecture and progenitor diversity while allowing controlled manipulation of signaling pathways, it enables quantitative testing of compounds that influence progenitor proliferation and differentiation. Serving as a bridge between stem-cell-derived cultures and in vivo models, this system can support preclinical evaluation of interventions such as EGFR modulation, growth factor combinations, or small molecules that enhance retinal repair and neurogenesis. Together, these findings establish a foundation for future single-cell transcriptomic analyses and functional assays to define the molecular trajectories and ultimate fates of the expanded progenitor populations.

## Data Availability

All data generated or analyzed during this study are included in this article. Further inquiries can be directed to the corresponding author.

## Supplementary Materials

The following supporting information can be downloaded at https://www.mdpi.com/article/10.3390/cells14231854/s1, Figure S1: Representative image of the distorted ONL organization in the EGF-treated REs. DAPI staining of the control (a) and EGF-treated REs (b) at 7 DIVs.
