# Supplementary figures and images for: Sustained EGFR Signaling Expands Otx2+ and Chx10+ Retinal Progenitors in the Postnatal Mouse Retina
Source: Cells. 2025 Nov 25;14(23):1854. doi: 10.3390/cells14231854 (PMC12691212; doi:10.3390/cells14231854)

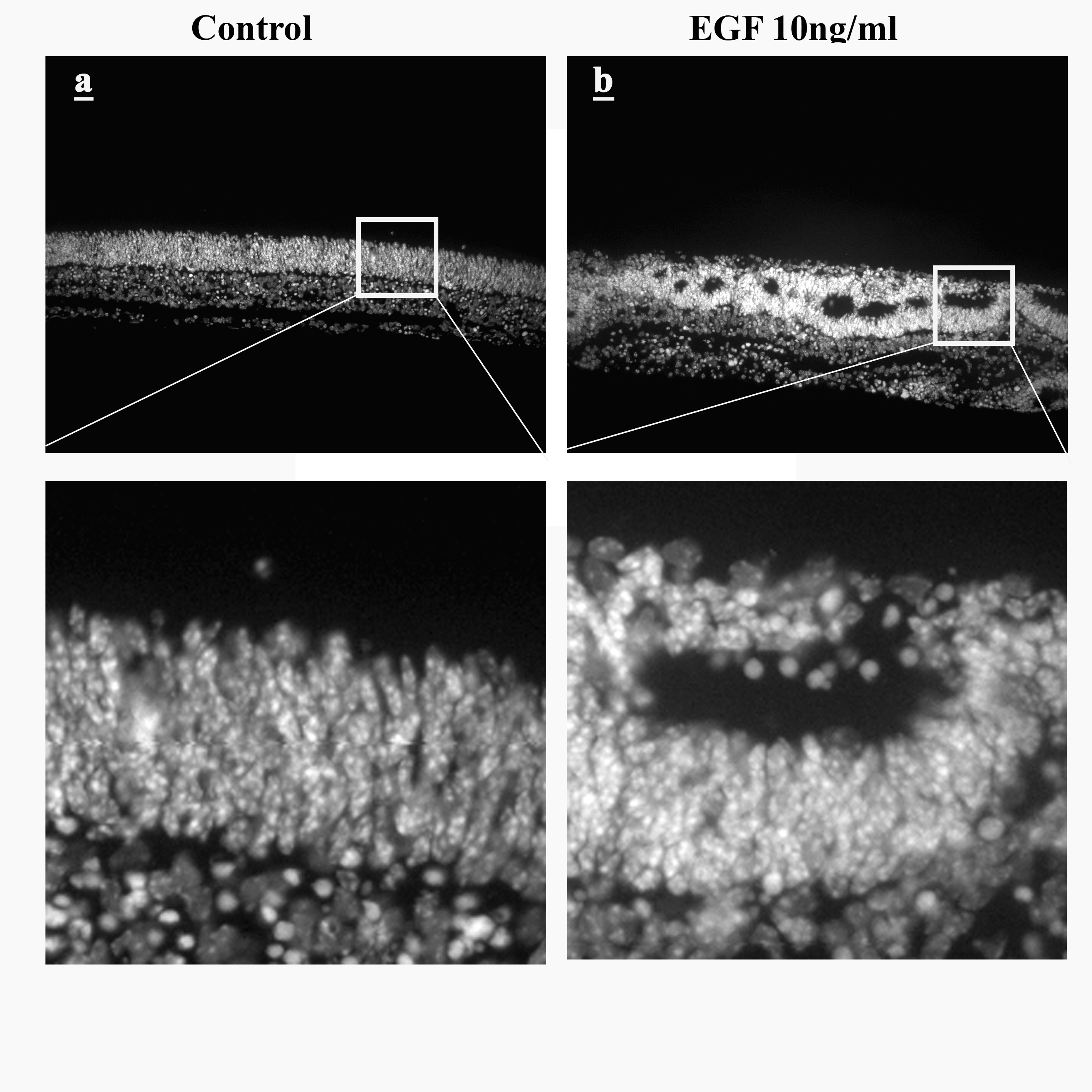

Supplement: Supplementary file 1 [file cells-14-01854-s001.zip › cells-3957348-supplementary s1.tiff]
